# Preclinical effects of *APOE* ε4 on cerebrospinal fluid Aβ42 concentrations

**DOI:** 10.1186/s13195-017-0313-3

**Published:** 2017-10-23

**Authors:** Ronald Lautner, Philip S. Insel, Tobias Skillbäck, Bob Olsson, Mikael Landén, Giovanni B. Frisoni, Sanna-Kaisa Herukka, Harald Hampel, Anders Wallin, Lennart Minthon, Oskar Hansson, Kaj Blennow, Niklas Mattsson, Henrik Zetterberg

**Affiliations:** 1 Department of Psychiatry and Neurochemistry, Institute of Neuroscience and Physiology, Sahlgrenska Academy at the University of Gothenburg, Gothenburg, Sweden; 2 Clinical Neurochemistry Laboratory, Sahlgrenska University Hospital, Mölndal, Sweden; 3 Clinical Memory Research Unit, Department of Clinical Sciences Malmö, Lund University, Lund, Sweden; 4 Department of Veterans Affairs Medical Center, Center for Imaging of Neurodegenerative Diseases, San Francisco, CA USA; 5 Department of Radiology and Biomedical Imaging, University of California, San Francisco, CA USA; 6 Department of Medical Epidemiology and Biostatistics, Karolinska Institutet, Stockholm, Sweden; 7 Istituto di Ricovero e Cura a Carattere Scientifico Centro San Giovanni di Dio Fatebenefratelli, Brescia, Italy; 8 Department of Neurology, University of Eastern Finland, Kuopio University Hospital, Kuopio, Finland; 9 AXA Research Fund and UPMC Chair, Sorbonne Universités, Université Pierre et Marie Curie (UPMC) Paris 06, Inserm, CNRS, Institut du cerveau et de la moelle (ICM), Département de Neurologie, Institut de la Mémoire et de la Maladie d’Alzheimer (IM2A), Hôpital Pitié-Salpêtrière, Boulevard de l’hôpital, F-75013 Paris, France; 10 Memory Clinic, Skåne University Hospital, Malmö, Sweden; 11 Department of Neurology, Skåne University Hospital, Lund, Sweden; 12 Department of Molecular Neuroscience, UCL Institute of Neurology, Queen Square, London, WC1N 3BG UK

**Keywords:** Alzheimer’s disease, *APOE*, Cerebrospinal fluid, Beta amyloid

## Abstract

**Background:**

From earlier studies it is known that the *APOE* ε2/ε3/ε4 polymorphism modulates the concentrations of cerebrospinal fluid (CSF) beta-amyloid_1–42_ (Aβ42) in patients with cognitive decline due to Alzheimer’s disease (AD), as well as in cognitively healthy controls. Here, in a large cohort consisting solely of cognitively healthy individuals, we aimed to evaluate how the effect of *APOE* on CSF Aβ42 varies by age, to understand the association between *APOE* and the onset of preclinical AD.

**Methods:**

*APOE* genotype and CSF Aβ42 concentration were determined in a cohort comprising 716 cognitively healthy individuals aged 17–99 from nine different clinical research centers.

**Results:**

CSF concentrations of Aβ42 were lower in *APOE* ε4 carriers than in noncarriers in a gene dose-dependent manner. The effect of *APOE* ε4 on CSF Aβ42 was age dependent. The age at which CSF Aβ42 concentrations started to decrease was estimated at 50 years in *APOE* ε4-negative individuals and 43 years in heterozygous *APOE* ε4 carriers. Homozygous *APOE* ε4 carriers showed a steady decline in CSF Aβ42 concentrations with increasing age throughout the examined age span.

**Conclusions:**

People possessing the *APOE* ε4 allele start to show a decrease in CSF Aβ42 concentration almost a decade before *APOE* ε4 noncarriers already in early middle age. Homozygous *APOE* ε4 carriers might deposit Aβ42 throughout the examined age span. These results suggest that there is an *APOE* ε4-dependent period of early alterations in amyloid homeostasis, when amyloid slowly accumulates, that several years later, together with other downstream pathological events such as tau pathology, translates into cognitive decline.

## Background

Alzheimer’s disease (AD) is a neurodegenerative disease characterized by the accumulation of extracellular beta-amyloid (Aβ) plaques and intracellular tau tangles [1]. AD pathology is multifactorial with both genetic and environmental risk factors, with the most prominent susceptibility gene being apolipoprotein E (*APOE*) [2]. The *APOE* gene is polymorphic, with three different alleles, of which the ε4 allele is associated with an increased risk, as well as a lower age at onset, of AD. Heterozygous *APOE* ε4 carriers have an approximately 3-fold increase of risk compared with individuals lacking the ε4 allele, whereas the increase of risk is up to 12-fold in homozygous *APOE* ε4 carriers [3]. The underlying pathophysiological mechanisms for this strong genetic association are still unknown, but may involve direct or indirect effects on Aβ aggregation or clearance [4, 5].

Measurement of the 42 amino acid isoform of Aβ (Aβ42) in the cerebrospinal fluid (CSF) is used alongside CSF total tau (T-tau) and CSF phosphorylated tau (P-tau) as a diagnostic tool for AD [6]. Decreased concentrations of CSF Aβ42 are indicative of cerebral amyloid pathology during the entire course of the disease, from preclinical asymptomatic disease to mild cognitive impairment (MCI) and dementia, and may even indicate disturbed amyloid metabolism before amyloid deposition may be visualized by amyloid PET imaging [7–9]. An association between the *APOE* genotype and CSF concentrations of Aβ42 has been described previously among patients with AD and MCI, as well as in healthy controls, with the *APOE* ε4 allele being associated with lower CSF Aβ42 concentrations in a gene dose-dependent manner [10–15]. However, because most studies have only analyzed older people, it is not clear whether this effect is present in all age groups irrespective of preexisting amyloid pathology, especially since an earlier study showed the effect to be absent in a small cohort of younger cognitively healthy individuals [14].

Consequently, the question that arises is at what age the potential effects of *APOE* ε4 on CSF Aβ42 can be detected. To tackle this question, we analyzed CSF Aβ42 as well as the *APOE* ε4 genotype in a large cohort consisting of 716 cognitively healthy individuals from 17 to 99 years of age. Specifically, we tested how CSF Aβ42 concentrations differ by age in the different *APOE* ε4 carrier groups.

## Methods

### Cohorts

The total cohort consisted of 716 cognitively healthy individuals from nine different centers in Sweden, Finland, Germany, and Italy with age ranging from 17 to 99 years. All centers were specialized memory clinics, except one, which is a psychiatry clinic specialized in affective disorders. All subjects underwent neurological examination as well as cognitive testing to exclude cognitive impairment. One of the subcohorts contained 138 patients with bipolar disorder, whereas the rest of the participants (*n* = 578) were healthy volunteers. Most study participants (except the bipolar disorder patients, who were recruited among patients at the specialized affective disorders clinic) were recruited by advertisement or among relatives or friends of patients who were evaluated on suspicion of cognitive dysfunction.

### Lumbar puncture

CSF samples were obtained by lumbar puncture in the L3/4 or L4/5 interspace, collected in polypropylene tubes, centrifuged, and stored frozen at –80 °C until analysis according to standard operating procedures [6]. The time frame during which samples were collected in each center in relation to the time of sample analysis was less than 5 years in all cohorts. Long-term stability of CSF Aβ42 at –80 °C has been evaluated in several studies [16–18], all of which show that CSF Aβ42 is stable at –80 °C. The majority of the biomarker analyses were performed at the Clinical Neurochemistry Laboratory at the Sahlgrenska University Hospital, Gothenburg, Sweden, but samples from Kuopio, Finland and Munich, Germany as well as from Italy were analyzed in local laboratories.

### CSF analyses

CSF Aβ42 concentrations were measured using a sandwich enzyme-linked immunosorbent assay (INNOTEST β-amyloid[1–42]; Fujirebio, Ghent, Belgium) designed to detect the 1st and 42nd amino acids in the Aβ protein as described previously [19]. A subset of the samples was analyzed using a multiplex semiautomated assay platform (xMAP Luminex AlzBio3; Fujirebio) as described previously [20]. All analyses were performed by experienced laboratory technicians who were blinded to all clinical information.

To adjust for variation in biomarker concentrations between the different laboratories, data were normalized by defining the largest center cohort as the reference group and then calculating factors between the *APOE* ε4-negative individuals from each participating center and the *APOE* ε4-negative individuals in the reference group. These factors were then applied to all data, hence relating biomarker concentrations in all of the different center cohorts to those in the reference group. There were no significant correlations between age and CSF Aβ42 concentrations in all but one of the subcohorts (in which the effect was minor, *r* ^2^ = –0.036, *P* = 0.037), which points toward a lack of a primary relation between these two parameters. Note that since the center cohort that was defined as the reference group used the xMAP Luminex AlzBio3 assay, the normalized concentrations of Aβ42 in this material were lower than the corresponding concentrations when using the INNOTEST β-amyloid[1–42] assay.

### *APOE* alleles

Genotyping for *APOE* (gene map locus 19q13.2) was performed using allelic discrimination technology (TaqMan; Applied Biosystems) or equivalent techniques. Genotypes were obtained for the two single nucleotide polymorphisms that define the ε2, ε3, and ε4 alleles.

### Statistical analysis

Comparisons of biomarker concentrations between *APOE* ε4 carrier groups were performed by one-way analysis of variance (ANOVA) for several independent samples. Comparisons of genotype frequencies between patients with bipolar disorder and healthy volunteers were performed using Pearson’s chi-squared test. Statistical significance was defined at *P* < 0.05 and all statistical calculations were performed using SPSS version 19 (SPSS Inc., Chicago, IL, USA).

The trajectory of CSF Aβ42 concentrations with respect to age in different *APOE* ε4 carrier groups was modeled using restricted cubic splines and ordinary least squares regression. The Akaike Information Criterion selected the optimal model to be estimated using three spline knots. Regression models included gender and the interaction between the two-parameter spline representation of age and *APOE* ε4 group and the main effects for age and *APOE* ε4 group. Age at the initial decline of CSF Aβ42 concentrations was taken to be the maximum Aβ42 concentration prior to a monotone descent with increasing age. Confidence intervals for age at initial decline were estimated using the margins (2.5 and 97.5 percentiles) of 500 bootstrap samples.

## Results

### Demographic, genetic, and biochemical data

The majority of individuals in the total cohort (70.7%) lacked the *APOE* ε4 allele, with 26.5% being heterozygous and 2.8% being homozygous *APOE* ε4 carriers (Table 1). The subcohort consisting of patients with bipolar disorder had similar *APOE* ε4 genotype frequency (*P* = 0.633) as well as similar concentrations of CSF Aβ42 (*P* = 0.302) compared to the healthy volunteers and was therefore pooled with the rest of the total cohort (data not shown). There were neither any gender differences with regards to *APOE* ε4 genotype frequency (*P* = 0.586) or CSF Aβ42 concentrations (*P* = 0.534). Table 2 presents detailed demographic and biochemical data for all of the subcohorts included in the analysis.

**Table 1:** Demographic, genetic, and biochemical data in the total cohort as well as divided into three age tertiles

	Total cohort (*n* = 716)	≤45 years (*n* = 237)	46–64 years (*n* = 242)	≥65 years (*n* = 237)
Demographic				
Age (years), mean (range)	53.3 (17–99)	29.9 (17–45)	57.3 (46–64)	72.6 (65–99)
Male, *n* (%)	305 (42.6)	114 (48.1)	104 (43.0)	87 (36.7)
Female, *n* (%)	411 (57.4)	123 (51.9)	138 (57.0)	150 (63.3)
Genetic				
*APOE* ε4^–/–^, *n* (%)	506 (70.7)	162 (68.4)	172 (71.1)	172 (72.6)
*APOE* ε4^+/–^, *n* (%)	190 (26.5)	69 (29.1)	64 (26.4)	57 (24.1)
*APOE* ε4^+/+^, *n* (%)	20 (2.8)	6 (2.5)	6 (2.5)	8 (3.4)
Biochemical: CSF Aβ42 (ng/L), mean (SD)				
All genotypes	252.1 (71.0)	251.9 (67.8)	264.9 (67.7)	239.2 (75.2)
*APOE* ε4^–/–^	261.2 (70.9)	257.2 (69.5)	274.5 (68.1)	251.8 (73.4)
*APOE* ε4^+/–^	234.8 (65.4)	241.4 (64.0)	248.1 (58.3)	211.9 (69.7)
*APOE* ε4^+/+^	185.5 (59.0)	231.3 (51.8)	167.8 (39.1)	164.4 (62.1)
*P* value*	<0.001	0.203	<0.001	<0.001

*P values indicate comparisons of CSF Aβ42 concentrations between the *APOE* ε4 carrier groups (for the total cohort as well as for each of the tertiles)

*APOE* apolipoprotein E, *Aβ42* beta-amyloid_1–__42_, *CSF* cerebrospinal fluid, *SD* standard deviation

**Table 2:** Demographic, genetic, and biochemical data in the individual subcohorts

	Total cohort (*n* = 716)	Cohort 1a (*n* = 138)	Cohort 1b (*n* = 72)	Cohort 2 (*n* = 81)	Cohort 3 (*n* = 121)	Cohort 4 (*n* = 39)	Cohort 5 (*n* = 37)	Cohort 6 (*n* = 48)	Cohort 7 (*n* = 58)	Cohort 8 (*n* = 24)	Cohort 9 (*n* = 44)	Cohort 10 (*n* = 54)
Location		Gothenburg, Sweden	Gothenburg, Sweden	Perugia, Italy	Stockholm, Sweden	Malmö, Sweden	Malmö, Sweden	Mölndal, Sweden	Kuopio, Finland	Munich, Germany	Stockholm, Sweden	Mölndal, Sweden
Type of clinic		Affective disorders clinic	Affective disorders clinic	Memory clinic	Memory clinic	Memory clinic	Memory clinic	Memory clinic	Memory clinic	Memory clinic	Memory clinic	Memory clinic
Diagnosis		Bipolar disorder	Controls	Controls	Controls	Controls	Controls	Controls	Controls	Controls	Controls	Controls
Age (years), mean (range)	53.3 (17–99)	39.4 (20–73)	37.9 (21–74)	53.0 (21–88)	68.0 (40–92)	72.4 (60–87)	62.9 (42–99)	66.6 (52–80)	65.6 (45–81)	63.0 (49–84)	60.9 (23–88)	22.0 (17–34)
Male, *n* (%)	305 (42.6)	55 (39.9)	27 (37.5)	22 (27.2)	36 (29.8)	15 (38.5)	16 (43.2)	22 (45.8)	30 (51.7)	15 (62.5)	19 (43.2)	48 (88.9)
Female, *n* (%)	411 (57.4)	83 (60.1)	45 (62.5)	59 (72.8)	85 (70.2)	24 (61.5)	21 (56.8)	26 (54.2)	28 (48.3)	9 (37.5)	25 (56.8)	6 (11.1)
*APOE* ε4^–/–^, *n* (%)	506 (70.7)	93 (67.4)	49 (68.1)	65 (80.2)	88 (72.7)	29 (74.4)	25 (67.6)	32 (66.7)	40 (69.0)	19 (79.2)	31 (70.5)	35 (64.8)
*APOE* ε4^+/–^, *n* (%)	190 (26.5)	41 (29.7)	20 (27.8)	14 (17.3)	32 (26.4)	10 (25.6)	10 (27.0)	15 (31.3)	16 (27.6)	5 (20.8)	11 (25.0)	16 (29.6)
*APOE* ε4^+/+^, *n* (%)	20 (2.8)	4 (2.9)	3 (4.2)	2 (2.5)	1 (0.8)	0 (0.0)	2 (5.4)	1 (2.1)	2 (3.4)	0 (0.0)	2 (4.5)	3 (5.6)
CSF Aβ42 assay and concentrations measured (ng/L), mean (SD)		xMAP Luminex AlzBio3	xMAP Luminex AlzBio3	INNOTEST β-amyloid[1–42]	INNOTEST β-amyloid[1–42]	INNOTEST β-amyloid[1–42]	xMAP Luminex AlzBio3	INNOTEST β-amyloid[1–42]	INNOTEST β-amyloid[1–42]	INNOTEST β-amyloid[1–42]	INNOTEST β-amyloid[1–42]	INNOTEST β-amyloid[1–42]
All genotypes	252.1 (71.0)	257.7 (57.7)	255.1 (54.2)	260.2 (91.5)	250.8 (75.8)	260.3 (67.6)	245.4 (58.7)	250.3 (39.8)	242.2 (71.9)	247.6 (72.3)	259.0 (92.4)	232.1 (84.6)
*APOE* ε4^–/–^	261.2 (70.9)	264.6 (57.2)	259.8 (51.7)	262.5 (93.9)	264.0 (68.2)	260.8 (66.0)	257.5 (66.2)	258.3 (36.8)	258.5 (75.0)	260.9 (72.9)	276.6 (89.1)	240.6 (93.0)
*APOE* ε4^+/–^	234.8 (65.4)	250.5 (51.4)	245.3 (63.1)	266.4 (76.0)	219.7 (82.5)	258.9 (75.7)	220.3 (29.0)	241.7 (31.4)	205.4 (52.2)	197.0 (46.3)	230.0 (90.4)	216.0 (66.6)
*APOE* ε4^+/+^	185.5 (59.0)	172.0 (67.9)	243.3 (21.1)	139.3 (24.6)	87.5 (N/A)	N/A	219.2 (13.4)	125.7 (N/A)	209.7 (10.0)	N/A	143.9 (31.5)	218.0 (75.1)

*APOE* apolipoprotein E, *Aβ42* beta-amyloid_1–42_, *CSF* cerebrospinal fluid, *SD* standard deviation, N/A not available

### CSF Aβ42 concentrations in relation to *APOE* genotype

In the total cohort, CSF Aβ42 concentrations were lower in *APOE* ε4 carriers than in noncarriers in a gene dose-dependent manner (*P* < 0.001, Table 1), which is in keeping with earlier findings [14]. However, when dividing the total cohort into tertiles according to age, the effect was present in the middle and upper tertiles among individuals aged 46 or older (*P* < 0.001, Table 1), whereas in the lower tertile, containing individuals aged 45 or younger, the difference was nonsignificant (*P* = 0.203, Table 1).

### CSF Aβ42 concentrations across different age groups

**Fig. 1:**
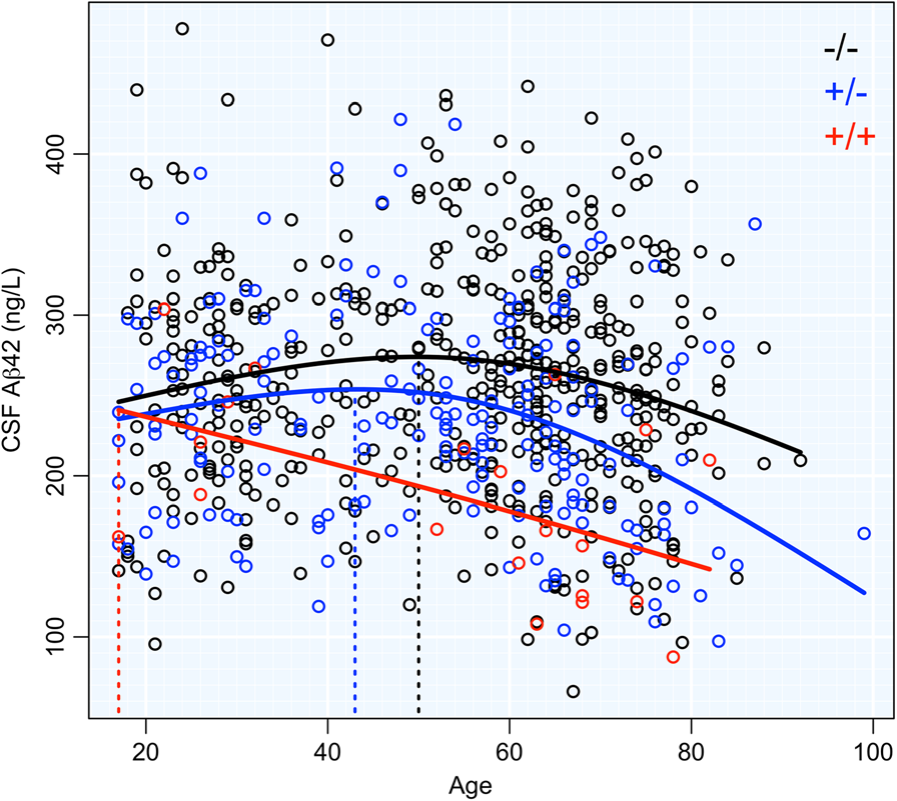
CSF Aβ42 concentrations plotted against age. Mean curves for the three *APOE* ε4 groups: black, *APOE* ε4^–/–^; blue, *APOE* ε4^+/–^; red, *APOE* ε4^+/+^. Vertical lines indicate age at initial decline of CSF Aβ42. *Aβ42* beta-amyloid_1–42_, *CSF* cerebrospinal fluid

The estimated curves showed an initial upslope of CSF Aβ42 concentrations in *APOE* ε4-negative individuals and heterozygous *APOE* ε4 carriers followed by a steep descent (Fig. 1). Aβ42 concentrations in homozygous *APOE* ε4 carriers, however, descended from an early age lacking the initial upslope. The age of initial descent, defined as the age at which CSF Aβ42 reaches its maximum, was estimated at 50 (95% confidence interval (CI) 42–54) years for *APOE* ε4-negative individuals and 43 (95% CI 17–48) years for heterozygous *APOE* ε4 carriers. This number could not be estimated in homozygous *APOE* ε4 carriers, as they lacked the initial upslope.

## Discussion

We conducted a large multicenter study to assess how effects of the *APOE* ε2/ε3/ε4 polymorphism on CSF Aβ42 concentrations vary by age in cognitively healthy individuals. The main findings were that: the *APOE* ε4 allele was associated with lower CSF Aβ42 concentrations overall in cognitively healthy people; the effects of *APOE* ε4 were present in older people but not in young people; and CSF Aβ42 started to decline at age 50 in people without the *APOE* ε4 allele, at age 43 in people carrying one *APOE* ε4 allele, and even earlier in people carrying two *APOE* ε4 alleles. Taken together, these findings show that *APOE* ε4 strongly modulates the effect of age on CSF Aβ42 in cognitively healthy people, and points to important age-dependent effects of *APOE* ε4 on the development of preclinical AD.

Comparisons of CSF Aβ42 and amyloid PET imaging in cognitively healthy people suggest that the first decline in CSF Aβ42 does not always translate to widespread cerebral amyloid deposition [7, 8, 21]. The age at which CSF Aβ42 concentrations start to decrease may therefore be the starting point for preclinical pathological disturbances in amyloid homeostasis, which ultimately results in amyloid accumulation that later becomes detectable on amyloid PET imaging. The data from this study suggest that the disturbed amyloid homeostasis occurs on average from age 50 in *APOE* ε4-negative individuals, and almost a decade earlier in *APOE* ε4 heterozygous people. In *APOE* ε4 homozygous people we estimated a descent in CSF Aβ42 already from age 17, but the sparsity of data among young homozygous *APOE* ε4 carriers makes this estimate uncertain, and we can only conclude that the decline in CSF Aβ42 starts considerably earlier in homozygous *APOE* ε4 carriers compared with heterozygous *APOE* ε4 carriers or noncarriers. Importantly, previous studies provide convergent evidence that emerging amyloid pathology, defined as decreased CSF Aβ42 concentrations, or CSF Aβ42 concentrations slightly above conventional thresholds for amyloid positivity, may have deleterious effects on brain structure, brain function, and cognition [22–25]. This highlights the importance of detecting the earliest effects of *APOE* ε4 on CSF Aβ42 in order to provide very early diagnostics and potentially initiate prevention of AD.

The fact that *APOE* ε4 affected CSF Aβ42 concentrations already from 43 years of age is interesting since a previous study found that *APOE* ε4 was associated with cognitive decline only after 50 years of age [26]. We therefore suggest that there is an intermediate period of early alterations in amyloid homeostasis before cognitive decline becomes detectable [23], when amyloid accumulation slowly builds up together with downstream pathological events (including spread of tau tangles), which ultimately translate to cognitive decline several years later.

This study has several limitations. First, we used cross-sectional data from several cohorts and several assays to measure CSF Aβ42. Although we employed normalization measures to bridge all results, the variability in cohorts and assays increases the variance of our models and estimates. Future studies are needed to verify these results in a monocenter setting, obviating the need for data normalization across cohorts. Second, the low number of *APOE* ε4 homozygous people, along with the sparsity of data in the age span between 85 and 100 years, limits our ability to model effects of *APOE* ε4 homozygosity and effects in the final part of the natural life span. Also, the lack of *APOE* ε4 homozygous people between age 35 and 50 makes it impossible to define whether there is a plateau in Aβ42 concentrations before decline or whether the concentrations drop directly from age 17 in homozygous *APOE* ε4 carriers.

## Conclusions

To sum up, the results of this study suggest that the process of preclinical Aβ pathology might start in early middle age in *APOE* ε4 carriers. Hence, we hypothesize that the *APOE* ε4 allele affects CSF Aβ42 concentrations by speeding up the process of preclinical Aβ accumulation and deposition in the brain. Studies addressing the molecular mechanisms behind the association between ApoE and cerebral Aβ build-up are needed to verify this.

## Abbreviations

AD: Alzheimer’s disease
ANOVA: Analysis of variance
*APOE*: Apolipoprotein E
Aβ42: Beta-amyloid_1–42_
CI: Confidence interval
CSF: Cerebrospinal fluid
MCI: Mild cognitive impairment
PET: Positron emission tomography
P-tau: Phosphorylated tau
T-tau: Total tau
